# Anthroposophic therapy for chronic depression: a four-year prospective cohort study

**DOI:** 10.1186/1471-244X-6-57

**Published:** 2006-12-15

**Authors:** Harald J Hamre, Claudia M Witt, Anja Glockmann, Renatus Ziegler, Stefan N Willich, Helmut Kiene

**Affiliations:** 1 Institute for Applied Epistemology and Medical Methodology, Böcklerstr. 5, 79110 Freiburg, Germany; 2 Institute of Social Medicine, Epidemiology, and Health Economics, Charité University Medical Center, Campus Mitte, 10098 Berlin, Germany; 3 Society for Cancer Research, Kirschweg 9, 4144 Arlesheim, Switzerland

## Abstract

**Background:**

Depressive disorders are common, cause considerable disability, and do not always respond to standard therapy (psychotherapy, antidepressants). Anthroposophic treatment for depression differs from ordinary treatment in the use of artistic and physical therapies and special medication. We studied clinical outcomes of anthroposophic therapy for depression.

**Methods:**

97 outpatients from 42 medical practices in Germany participated in a prospective cohort study. Patients were aged 20–69 years and were referred to anthroposophic therapies (art, eurythmy movement exercises, or rhythmical massage) or started physician-provided anthroposophic therapy (counselling, medication) for depression: depressed mood, at least two of six further depressive symptoms, minimum duration six months, Center for Epidemiological Studies Depression Scale, German version (CES-D, range 0–60 points) of at least 24 points. Outcomes were CES-D (primary outcome) and SF-36 after 3, 6, 12, 18, 24, and 48 months. Data were collected from July 1998 to March 2005.

**Results:**

Median number of art/eurythmy/massage sessions was 14 (interquartile range 12–22), median therapy duration was 137 (91–212) days. All outcomes improved significantly between baseline and all subsequent follow-ups. Improvements from baseline to 12 months were: CES-D from mean (standard deviation) 34.77 (8.21) to 19.55 (13.12) (p < 0.001), SF-36 Mental Component Summary from 26.11 (7.98) to 39.15 (12.08) (p < 0.001), and SF-36 Physical Component Summary from 43.78 (9.46) to 48.79 (9.00) (p < 0.001). All these improvements were maintained until last follow-up. At 12-month follow-up and later, 52%–56% of evaluable patients (35%–42% of all patients) were improved by at least 50% of baseline CES-D scores. CES-D improved similarly in patients not using antidepressants or psychotherapy during the first six study months (55% of patients).

**Conclusion:**

In outpatients with chronic depression, anthroposophic therapies were followed by long-term clinical improvement. Although the pre-post design of the present study does not allow for conclusions about comparative effectiveness, study findings suggest that the anthroposophic approach, with its recourse to non-verbal and artistic exercising therapies can be useful for patients motivated for such therapies.

## Background

Depressive disorders are a major health problem, affecting one-fourth to one-third of women and one-sixth of men at some time in life [1]. Every tenth patient seen in primary care has a depressive disorder, but in half of these patients, the depression is not diagnosed by the physician [2].

In Europe, depressive disorders are the third leading cause of disability [3]. Compared to the general population, Major Depression sufferers have a 20-fold increased risk of suicide [4]. Depressive disorders are also associated with increased morbidity and mortality from somatic diseases, including coronary heart disease [5].

Standard treatment for depression is antidepressant drugs and/or psychotherapy. Even under the optimum conditions of a clinical trial, half of included patients will not respond to newer antidepressants [6] and up to two-thirds of patients enrolled for psychotherapy will either not complete treatment or not respond to it [7].

Furthermore, evidence from randomised trials of antidepressants (and psychotherapy) does not apply to the 86% (and 68%) of patients with clinical features leading to study exclusion [7,8]. Thus, for a large proportion of depression patients, standard therapy remains unsatisfactory or is not evaluated.

Anthroposophic medicine (AM) was founded in the 1920s by Rudolf Steiner and Ita Wegman [9]. AM is provided by physicians and non-medical therapists in 67 countries worldwide [10]. AM acknowledges a spiritual-existential dimension in man which is assumed to interact with psychological and somatic levels in health and disease. AM therapy for depression aims to counteract constitutional vulnerability, stimulate salutogenetic self-healing capacities, and strengthen patient autonomy [11].

The AM approach differs from ordinary treatment in the use of non-verbal artistic and physical therapies [12-16] and special AM medication [17,18], and in the existentialist and biographical outlook of AM-inspired counselling and psychotherapy [19,20]. Similar to recent guideline recommendations [21], conventional antidepressant drugs are not used as initial therapy for mild depression. In severe depression, however, AM therapies are often combined with antidepressants [17].

In AM art therapy (AAT) patients engage in painting, drawing, clay modelling, music or speech exercises [22]. In addition to psychological effects (e.g. activation, emotive expression, dialogical communication with the therapist and with the artistic medium [23,24]), AAT can induce physiological effects: e.g. AAT speech exercises have effects on heart rate rhythmicity and cardiorespiratory synchronisation which are not induced by spontaneous or controlled breathing alone [25,26].

AM eurythmy therapy (EYT, Greek "harmonious rhythm") is an active exercise therapy, involving cognitive, emotional, and volitional elements [27]. During EYT sessions patients are instructed to perform specific movements with the hands, the feet or the whole body. EYT movements are related to the sounds of vowels and consonants, to music intervals or to soul gestures, e.g. sympathy-antipathy. Between therapy sessions patients practice eurythmy movements daily [28]. EYT is assumed to have both general effects (e.g. improving breathing patterns and posture, strengthening muscle tone, enhancing physical vitality [11]) and specific therapeutic effects.

AM rhythmical massage therapy (RMT) was developed from Swedish massage [13] by Ita Wegman, physician and physiotherapist. In RMT, traditional massage techniques (effleurage, petrissage, friction, tapotement, vibration) are supplemented by gentle lifting and rhythmically undulating, stroking movements, where the quality of grip and emphasis of movement are altered to promote specific effects [11,29,30].

AM medications are of mineral, botanical or zoological origin, and are mostly used in homeopathic dilutions [31].

Small observational studies found positive effects from AM therapy components in depressed inpatients [15,16,32]. Here we present a study of comprehensive AM therapy for depression in outpatient settings.

## Methods

### Study design and objective

This is a prospective four-year cohort study in a real-world medical setting. The study was initiated by a health insurance company and was part of a research project on the effectiveness and costs of AM therapies in outpatients with chronic disease (Anthroposophic Medicine Outcomes Study, AMOS [33]). The primary research question was: Is AM therapy of outpatients with depression associated with clinically relevant improvement of depressive symptoms? Further research questions concerned health status, use of adjunctive therapies, adverse reactions, and therapy satisfaction.

### Setting, participants, and therapy

Participating physicians were certified by the Physicians' Association for Anthroposophical Medicine in Germany and had office-based practice or worked in outpatient clinics in Germany. Participating AM therapists were certified by the Association for Anthroposophical Art Therapy in Germany (AAT), the Eurythmy Therapy Association of Germany (EYT), and the German Rhythmical Massage Therapy Association (RMT), respectively.

The physicians were instructed to recruit consecutive outpatients fulfilling the eligibility criteria. Inclusion criteria were (1) Age 17–70 years, (2) depressed mood plus at least two of the following symptoms (DSM-IV symptoms of dysthymic disorder): poor appetite or overeating, insomnia or hypersomnia, low energy or fatigue, low self-esteem, poor concentration or difficulty making decisions, feelings of hopelessness, (3) symptom duration ≥ six months, (4) Center for Epidemiological Studies Depression Scale, German version (CES-D) of at least 24 points, (5) starting AM therapy for depressive symptoms: referral to AM therapist (AAT, EYT or RMT) or starting AM therapy provided by study physician (MED: AM-related consultations, AM medication) after an initial AM-related consultation of at least 30 min. Exclusion criteria were previous AM therapy (AAT, EYT, RMT, or previous AM-related consultation of at least 30 min, respectively) for depression.

The decision to start AM therapy for depression was part of the AM physicians' routine clinical practice. Patients were treated according to the physicians' and therapists' discretion. AM therapies (AAT, EYT, RMT, and MED) were evaluated as a therapy package; other therapies, including psychotherapy and antidepressants, were evaluated as non-AM adjunctive therapies.

### Clinical outcomes

• CES-D (primary outcome [34]) from 0 ("no depressive symptoms") to 60 ("maximum symptoms"). Patients document the frequency of 20 symptoms during the last week, from 0 ("rarely or none of the time ≈ less than 1 day") to 3 ("most or all of the time ≈ 5–7 days"). The German version [35] classifies persons with a score ≥ 24 points as depressed.

• Health status: SF-36 [36] Mental and Physical Component Summary Measures, eight scales, and Health Change item.

• Disease severity on numerical rating scales [37] from 0 („not present“) to 10 („worst possible“): Disease Score (physician's global assessment, documented in patients enrolled up to 30 Sep 2000); Symptom Score (patients' assessment of one to six most relevant symptoms present at baseline, documented in patients enrolled after 1 Jan 1999).

Disease Score was documented after 0, 6, and 12 months, other outcomes after 0, 3, 6, 12, 18, 24, and 48 months.

### Other outcomes

• Therapy ratings after six and 12 months: Patient rating of therapy outcome, patient satisfaction with therapy, AAT/EYT/RMT effectiveness rating by patient and physician.

• Adverse drug or therapy reactions during the first 24 study months: cause, intensity (mild/moderate/severe = no/some/complete impairment of normal daily activities, respectively); Serious Adverse Events (physician and patient documentation).

### Data collection

All data were documented with questionnaires sent in sealed envelopes to the study office. Physicians documented inclusion criteria No 2 and 3 and AM-related consultations; therapists documented AAT/EYT/RMT administration; all other items were documented by patients, unless otherwise stated. Patient responses were not made available to physicians. Physicians were compensated €40 per included and fully documented patient, while patients received no compensation.

Data were entered twice by two different persons into Microsoft^® ^Access 97. The two datasets were compared and discrepancies resolved by checking with the original data.

### Quality assurance, adherence to regulations

The study was approved by the Ethics Committee of the Faculty of Medicine Charité, Humboldt University Berlin, and was conducted according to the Helsinki Declaration and the ICH-GCP guidelines. Written informed consent was obtained from all patients before enrolment.

### Data analysis

The data analysis (SPSS^® ^13.0.1, StatXact^® ^5.0.3) was performed on all patients fulfilling eligibility criteria. Clinical outcomes were analysed in patients with evaluable data for each follow-up, without replacement of missing values. For continuous data the Wilcoxon Signed-Rank test was used for paired samples and the Mann-Whitney U-test for independent samples, median group differences with 95% confidence interval (95%-CI) were estimated according to Hodges and Lehmann [38]. For binominal data McNemar test and Fisher's exact test were used. All tests were two-tailed. Significance criteria were p < 0.05 and 95%-CI not including 0. Pre-post effect sizes were calculated as Standardised Response Mean and classified as small (0.20–0.49), medium (0.50–0.79), and large (≥ 0.80) [39].

## Results

### Participating physicians and therapists

59 physicians screened patients. 42 physicians included patients into the study; these physicians did not differ significantly from all AM-certified physicians in Germany (n = 362) regarding gender (57.1% vs. 62.2% males), age (mean 45.9 ± 7.0 vs. 47.5 ± 7.9 years), number of years in practice (18.8 ± 7.3 vs. 19.5 ± 8.7 years), or the proportion of primary care physicians (90.5% vs. 85.0%). Patients were treated by 52 AAT/EYT/RMT therapists (AAT: n = 24, EYT: n = 23, RMT: n = 5). Comparing these therapists to certified therapists without study patients (n = 706; AAT: n = 230, EYT: n = 326, RMT: n = 150), no significant differences were found regarding gender (82.7% vs. 79.3% females), age (mean 48.4 ± 6.9 vs. 51.3 ± 9.6 years), or median number of years since graduation from AAT school (15.0 years, interquartile range IQR 10.0–18.0 vs. 14.0 years, IQR 11.0–19.0) or EYT school (10.0 years, IQR 7.0–14.0 vs. 12.0 years, IQR 8.0–20.0).

### Patient recruitment and follow-up

**Figure 1:**
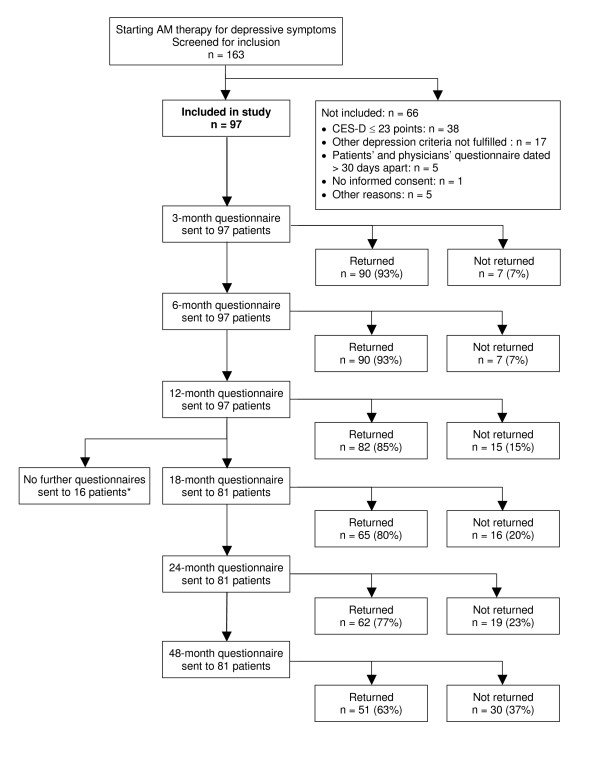
**Patient recruitment and follow-up**. *18-, 24-, and 48-month follow-up questionnaires were not sent to patients enrolled before 1 Jan 1999.

From 1 July 1998 to 31 March 2001, a total of 163 patients starting AM therapy for depressive symptoms were screened for inclusion. 97 patients fulfilled all eligibility criteria and were included in the study (Figure 1). The last patient follow-up ensued on 30 March 2005. Included and not included patients (n = 66) did not differ significantly regarding age, gender, duration of the depressive disorder, baseline Disease Score, or baseline Symptom Score. Most frequent reason for non-inclusion was non-fulfilment of depression severity criteria (CES-D ≤ 23 points, n = 38/66); baseline CES-D score was median 34.0 (IQR 28.0–38.0) points in included and 18.0 (IQR 14.3–23.8) points in not included patients (median difference 15.0 points, 95%-CI 12.0–18.0, p < 0.01).

The number of depression patients eligible for screening (referred to AAT/EYT/RMT or starting MED for depressive symptoms) during the recruitment period is not known, but the total number of patients referred to AAT/EYT/RMT in the AMOS project, regardless of diagnosis, was estimated by the physicians (response rate 62.2%, 74/119 physicians). The proportion of referred vs. enrolled patients was median 3.9 (IQR 0.5–10.0). There was no correlation between this proportion and the 0–12 month improvement of Symptom Score (Spearman-Rho -0.04, p = 0.496, n = 364 patients).

92% (89/97) of patients were enrolled by altogether 38 primary care physicians (35 general practitioners and three internists), 8% (8/97) were enrolled by four physicians in referral practices or outpatient clinics (two internists, two physicians with psychotherapy qualification).

98% (95/97) of patients returned at least one follow-up questionnaire, 2% (2/97) had no follow-up data. The 12-month questionnaire was returned by 85% of patients; these patients did not differ significantly from non-respondents (15%) regarding age, gender, duration of depressive disorder, or baseline CES-D. Corresponding dropout analyses for 24-month follow-up also showed no differences. The physician follow-up documentation was available for 86% (83/97) of patients after six months and for 85% after 12 months.

### Baseline characteristics

Median duration of the depressive disorder was 5.0 (IQR 2.0–10.0) years. 80% (78/97) of patients had a current comorbid disease, median 2.0 (IQR 1.0–3.0) diseases per patient. Most common comorbid diseases, classified by ICD-10, were M00-M99 Musculoskeletal Diseases (24.6%, 46/187 diagnoses), E00–E90 Endocrine, Nutritional and Metabolic Diseases (14.4%), and F00–F99 Mental Disorders (9.6%). 18% (17/97) of patients had a current or previous comorbid mental disorder, 24% (23/97) had a history of inpatient psychiatric treatment.

The patients were recruited from 13 of 16 German federal states. Mean age was 42.9 ± 9.9 years (range 20–69 years).

Compared to the German population, the patients had higher educational and occupational levels, had less daily alcohol consumers and regular smokers, and were less overweight; the patients' socio-demographic status was similar to the population regarding low-income, living alone, and sport; and less favourable for work disability pension, severe disability status, and sick-leave (Table 1).

**Table 1:** Baseline data of study population

Items	Study patients			German primary care patients	
	
		N	%	%	Source
Female gender		82/97	85%	51%	[57]
Age groups	20–29 years	9/97	9%	12%	
	30–49 years	67/97	69%	32%	[57]
	50–69 years	21/97	22%	23%	
Duration of depressive disorder	6–11 months	10/97	10%		
	12–23 months	7/97	7%		
	≥ 24 months	80/97	82%		

	Study patients enrolled after 1. Jan 1999			German population	

"Fachhochschule" or university entrance qualification		37/81	46%	19%	[58]
University degree		13/81	16%	6%	[58]
Wage earners		3/81	4%	18%	[58]
Unemployed during last 12 months	Economically active patients	9/70	13%	10%	[58]
Living alone		16/81	20%	21%	[58]
Net family income < 900 € per month		11/65	17%	16%	[58]
Alcohol use daily (patients) vs. almost daily (Germany)	Male	0/10	0%	28%	[59]
	Female	1/71	1%	11%	
Regular smoking	Male	2/10	20%	37%	[60]
	Female	11/71	15%	28%	
Sports activity ≥ 1 hour weekly	Age 25–69	38/79	48%	39%	[61]
Body mass index < 18.5 (low)	Male	0/10	0%	1%	[62]
	Female	5/70	7%	4%	
Body mass index ≥ 25 (overweight)	Male	0/10	0%	56%	[62]
	Female	22/70	31%	39%	
Permanent work disability pension		19/81	15%	3%	[63]
Severe disability status		24/81	30%	12%	[64]
Sick leave days in the last 12 months (mean ± SD)	Economically active patients	45 ± 82		17.0	[65]

### Therapies

At study entry, the patients started MED therapy (n = 13) or were referred to AM therapies (n = 84, thereof AAT: n = 42, EYT: n = 36, RMT: n = 6). AAT techniques were painting/drawing/clay (n = 28), speech exercises (n = 12), and music (n = 2). AAT/EYT/RMT was definitely administered to 98% (82/84) of patients and started median 8 (IQR 0–28) days after enrolment. Median therapy duration was 137 (IQR 91–212) days, median number of therapy sessions was 14 (IQR 12–22, mean 16.8 ± 9.5). During the first study year, the patients had median 1.0 (IQR 0.0–4.0, range 0–30) AM-related consultation with their study physician; 77% (66 of 86 evaluable patients) used AM medication, with a median of 0.43 (IQR 0.02–1.43) AM medications per day throughout the first study year.

In the first six study months 29% (n = 24/84) of evaluable patients used antidepressants (ATC-Index N06A or hypericum preparations) for at least six days, 24% (n = 20/84) had at least ten psychotherapy sessions, whereas 55% (n = 46/84) used neither psychotherapy nor antidepressants.

### Clinical outcomes

**Figure 2:**
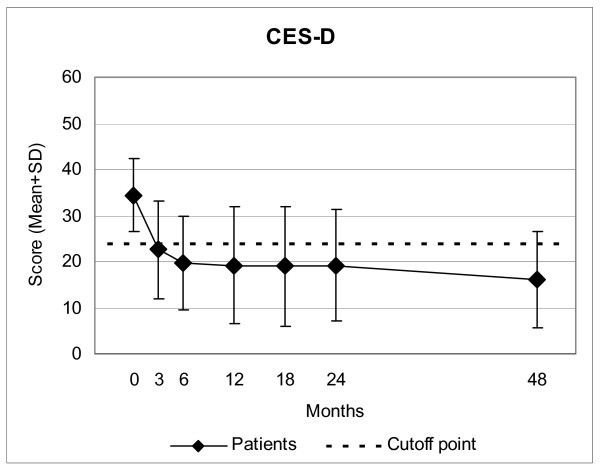
**Center for Epidemiological Studies Depression Scale (CES-D), German version**. Higher scores indicate more depressive symptoms. Cutoff point: Subjects with a score of ≥ 24 points are classified as depressed.

CES-D, Symptom and Disease Scores, and all eleven SF-36 scores improved significantly between baseline and all subsequent follow-ups. For all these 14 outcomes, the most pronounced improvement occurred during the first six months; improvements were maintained until the last follow-up (Figure 2 to Figure 4). Effect sizes for the 0–12-month comparison were large (range 0.80–1.77) for 11 outcomes and medium (0.54–0.76) for three outcomes (Table 2). All these improvements remained stable until the last follow-up.

**Figure 3:**
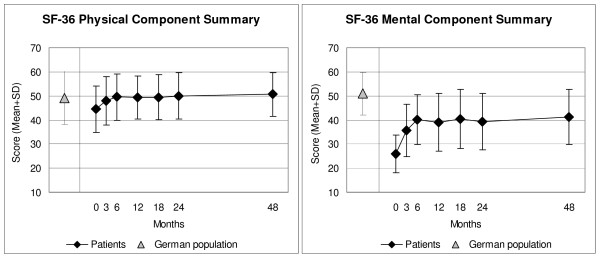
**SF-36 Physical and Mental Component Summary Measures**. Higher scores indicate better health. Adult patients and German population (age 17–74 years) [36].

**Figure 4:**
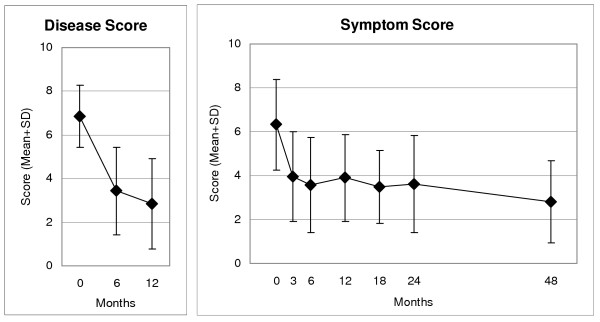
**Disease Score (physicians' assessment), Symptom Score (patients' assessment)**. 0 "not present", 10 "worst possible"

**Table 2:** Clinical outcomes 0–12 months

Item	N	0 months	12 months	P-value	Median difference (95%-CI)*	Improved**	SRM
						
		Mean (SD)	Mean (SD)				
CES-D (0–60)							
-all patients	75	34.77 (8.21)	19.55 (13.12)	p < 0.001	15.50 (12.50–18.50)	85%	1.20
-AM Art Therapy	33	36.94 (8.58)	21.12 (11.99)	p < 0.001	15.50 (11.50–19.50)	91%	1.32
--painting/drawing/clay	27	38.95 (8.24)	23.18 (12.52)	p < 0.001	15.00 (10.50–21.50)	91%	1.25
-Eurythmy Therapy	27	30.70 (5.55)	16.67 (13.32)	p < 0.001	15.00 (8.50–19.50)	81%	1.08
Disease Score (0–10)	57	7.09 (1.38)	2.84 (2.07)	p < 0.001	4.50 (4.00–5.00)	95%	1.77
Symptom Score (0–10)	69	6.35 (1.47)	3.90 (2.42)	p < 0.001	2.63 (1.92–3.29)	81%	0.91
SF-36 Mental Component	80	26.11 (7.98)	39.15 (12.08)	p < 0.001	13.04 (10.45–16.05)	83%	1.11
SF-36 Physical Component	80	43.78 (9.46)	48.79 (9.00)	p < 0.001	4.96 (3.02–6.84)	71%	0.59
SF-36 Scales (0–100)							
Physical Function	82	75.12 (22.80)	85.15 (19.00)	p < 0.001	10.00 (5.00–12.50)	63%	0.54
Role Physical	81	31.58 (35.52)	68.21 (38.33)	p < 0.001	50.00 (37.50–62.50)	65%	0.88
Role-Emotional	80	22.92 (32.52)	60.21 (39.38)	p < 0.001	50.00 (33.34–66.67)	69%	0.87
Social Functioning	82	43.14 (22.92)	65.70 (26.48)	p < 0.001	25.00 (18.75–37.50)	72%	0.80
Mental Health	81	33.48 (13.82)	56.10 (19.05)	p < 0.001	22.00 (18.00–28.00)	88%	1.19
Bodily Pain	82	50.27 (26.74)	66.67 (25.06)	p < 0.001	19.50 (12.50–26.00)	63%	0.65
Vitality	81	23.50 (12.47)	46.05 (19.47)	p < 0.001	25.00 (20.00–30.00)	68%	1.22
General Health	81	41.80 (18.55)	55.81 (20.35)	p < 0.001	13.50 (10.00–18.50)	78%	0.76
SF-36 Health Change***	81	3.58 (1.00)	2.10 (1.08)	p < 0.001	2.00 (1.50–2.00)	63%	1.06

*Positive differences indicate improvement. **Percentage of patients improved from baseline. ***SF-36 Health Change: range from 1 ("much better now than one year ago") to 5 ("much worse now than one year ago"). SRM: Standardised Response Mean effect size (small: 0.20–0.49, medium: 0.50–0.79, large: ≥ 0.80)

CES-D improved progressively at each follow-up except between 12 and 18 months after study entry (Figure 2). At 12-month follow-up and later, 52%–56% of evaluable patients (35%–42% of all patients) were improved by at least 50% of baseline CES-D scores; 66%–77% of evaluable patients (47%–52% of all patients) were no longer classified as depressed (CED-D = 23 points) (Table 3). The CES-D improvements were similar in patients receiving EYT and AAT, and in the AAT subgroup using painting/drawing/clay techniques (Table 2).

**Table 3:** CES-D: Responder rates at follow-ups

Follow-up month	CES-D improved from baseline		CES-D improved from baseline by ≥ 50%		CES-D ≤ 23 (not depressed)	
	
	Proportion of evaluable patients	Proportion of all patients	Proportion of evaluable patients	Proportion of all patients	Proportion of evaluable patients	Proportion of all patients
	N (%)	N (%)	N (%)	N (%)	N (%)	N (%)

3	59/67 (88%)	59/79 (75%)	21/67 (31%)	21/79 (27%)	39/67 (58%)	39/79 (49%)
6	60/69 (87%)	60/79 (79%)	27/69 (39%)	27/79 (34%)	47/69 (68%)	47/79 (59%)
12	54/62 (87%)	54/79 (68%)	32/62 (52%)	32/79 (41%)	41/62 (66%)	41/79 (52%)
18	50/59 (85%)	50/79 (63%)	33/59 (56%)	33/79 (42%)	41/59 (69%)	41/79 (52%)
24	54/60 (90%)	54/79 (68%)	31/60 (52%)	31/79 (41%)	40/60 (67%)	40/79 (51%)
48	43/48 (90%)	43/79 (54%)	28/48 (56%)	28/79 (35%)	37/48 (77%)	37/79 (47%)

Patients enrolled after 1.1.99. CES-D: Center for Epidemiological Studies Depression Scale, German version

In order to estimate the influence of four bias factors on 0–12 month CES-D outcomes we performed post-hoc sensitivity analyses. The first sensitivity analysis (Table 4, SA1) concerned dropout bias. The main analysis had comprised all enrolled patients with evaluable CES-D data at baseline and 12-month follow-up. In the first sensitivity analysis, missing values after 12 months were replaced with the last value carried forward, reducing the average 0–12 month CES-D improvement by 9% (15.23→13.90 points). The second analysis (Table 4, SA2) concerned natural recovery, which is unlikely in depression of more than 1–2 years duration [40-45]: The sample was restricted to patients with a duration of the depressive disorder of at least 2 years, reducing the improvement by 8% (15.23→14.05 points). The third analysis (Table 4, SA3) concerned the effects of relevant adjunctive therapies: The sample was restricted to patients using neither psychotherapy nor antidepressants in the first six study months (see "Therapies" above). In these patients the CES-D showed a similar improvement to that of all study patients (15.29 vs. 15.22 points). The fourth analysis (Table 4, SA4) concerned regression to the mean resulting from extreme group selection due to the inclusion criterion of CES-D ≥ 24: The sample was extended to also include screened patients starting AM therapy for depressive symptoms but not fulfilling all depression criteria for study inclusion. This extension of the eligibility criteria lead to a reduction of the average 0–12 month CES-D improvement by 27% (analysis of patients enrolled after 1 Jan 2000: 13.38→9.82 points). Combining SA1+SA2+SA4, the improvement was reduced by altogether 35% (13.38→8.64 points) but still remained significant.

**Table 4:** CES-D: Sensitivity analysis (SA) of 0–12 month outcomes

Analysis	N	0 months		12 months		0–12 month difference			
		
		Mean	SD	Mean	SD	Mean	SD	P-value	Median difference (95%-CI)
**All patients enrolled in depression study**									
Main analysis: enrolled patients with evaluable data at 0 and 12 months	75	34.77	(8.21)	19.55	(13.12)	15.23	(12.72)	p < 0.001	15.50 (12.50–18.50)
SA1: last value carried forward	93	34.67	(7.95)	20.77	(12.56)	13.90	(12.29)	p < 0.001	15.00 (12.00–17.50)
SA2: Patients with a disease duration of ≥ 24 months	61	35.10	(8.25)	21.05	(13.53)	14.05	(13.07)	p < 0.001	14.50 (10.50–17.50)
SA3 Patients not using antidepressants or psychotherapy	39	33.03	(6.98)	17.74	(12.50)	15.29	(10.80)	p < 0.001	15.50 (12.00–19.00)
SA1+SA2+SA3	33	33.27	(6.88)	19.33	(13.16)	13.94	(11.85)	p < 0.001	14.00 (10.00–18.00)
AMOS patients screened for depression study after 1 Jan 2000*									
Main analysis: enrolled patients with evaluable data at 0 and 12 months	37	33.65	(7.64)	20.27	(13.07)	13.38	(11.98)	p < 0.001	14.50 (9.50–17.50)
SA4: all screened patients (not enrolled + enrolled) with evaluable data at 0 and 12 months	57	28.35	(11.25)	18.53	(12.36)	9.82	(13.53)	p < 0.001	10.00 (6.50–14.00)
SA1+SA2+SA4	58	29.36	(10.51)	20.72	(12.03)	8.64	(12.46)	p < 0.001	9.50 (6.00–13.00)

CES-D: Center for Epidemiological Studies Depression Scale, German version. AMOS: Anthroposophic Medicine Outcomes Study. *12-month follow-up documentation of CES-D was not performed for AMOS patients enrolled before 1 Jan 2000, except for patients also enrolled into depression study. **Percentage of patients improved from baseline. SRM: Standardised Response Mean effect size (small: 0.20–0.49, medium: 0.50–0.79, large: ≥ 0.80)

### Other outcomes

At six-month follow-up, the patients' average therapy outcome rating (numeric scale: 0 "no help at all", 10 "helped very well") was 7.54 ± 1.76; patient satisfaction with therapy (0 "very dissatisfied", 10 "very satisfied") was 7.92 ± 1.86. The patients' AAT/EYT/RMT effectiveness rating was positive ("very effective" or "effective") in 88% (66/75) of patients, and negative ("less effective", "ineffective" or "not evaluable") in 12%. The physicians' effectiveness rating was positive in 78% (56/72) and negative in 22%. The ratings of therapy outcome, satisfaction, and effectiveness did not differ significantly between six- and 12-month follow-ups.

During the first 24 study months adverse reactions to AAT/EYT/RMT were reported in one patient (repeated loss of voice after AAT singing therapy, moderate intensity), adverse reactions to AM medication in two patients (moderate dizziness from Geum urbanum – not medically confirmed; mild nausea from Choleodoron – medically confirmed); none of these reactions led to therapy discontinuation. Adverse reactions to non-AM medication were reported in 12 patients (antidepressants: n = 5, other psychoactive drugs: n = 2, other medication: n = 5; medication stopped in three patients). One patient had adverse reactions (pain) to psychotherapy, which was stopped.

One Serious Adverse Event (SAE) occurred: A 53-year old woman was acutely hospitalised because of perforation of the small intestine after swallowing fish bones. She recovered completely. This SAE had no relation to any therapy or medication.

## Discussion

This prospective cohort study is the first study of comprehensive AM therapy for depression, and the first depression study conducted in outpatient AM settings. The study was conducted in conjunction with a health insurance program and aimed to provide information on AM use under routine conditions in Germany. We studied adult outpatients starting AM therapies for depression (depressed mood + at least two of six further depressive symptoms + CES-D ≥ 24). Following AM therapies, substantial improvements of symptoms and health status (SF-36) were observed. Improvements were maintained during the four-year follow-up. Adverse reactions to AM therapy or medication were infrequent (2% of patients), of mild-to-moderate intensity, and did not require therapy discontinuation.

### Strengths and limitations

Strengths of this study include a long follow-up period, high follow-up rates, and the participation of 8% of all AM-certified physicians and AM therapists in Germany. The participants resembled all eligible physicians/therapists with respect to socio-demographic characteristics, and included patients resembled not included, screened patients regarding baseline characteristics. These features suggest that the study to a high degree mirrors contemporary AM practice. Nevertheless, since the study had a long recruitment period, the participating physicians were not able to screen all their eligible patients (patients starting AM therapy for depressive symptoms). For the whole AMOS project it was estimated that the physicians enrolled every fourth patient referred to AAT/EYT/RMT. Selection bias could be present if physicians would preferentially screen and enrol such patients for whom a particularly positive outcome was expected. In this case one would expect the degree of selection (= the proportion of referred vs. enrolled patients) to correlate positively with clinical outcomes. That was not the case, the correlation was almost zero (-0.04). This analysis of AAT/EYT/RMT patients (87% of the present cohort) does not suggest that physicians' screening of patients starting AM therapy was affected by selection bias.

An important limitation of the study is the absence of a comparison group receiving another treatment or no therapy. For this reason we tried as far as possible to assess the influence of other causes apart from the AM therapies. Sensitivity analyses were conducted in regard to regression to the mean due to extreme group selection (CES-D ≥ 24 points at inclusion), spontaneous improvement and dropout bias. According to the analyses, these three factors can together explain maximum 35% of the average 0–12-month improvement. Notably, this analysis does not completely exclude regression to the mean due to symptom fluctuation with preferential self-selection to therapy and study inclusion at symptom peaks. Another form of self-selection bias is also possible, since a prerequisite for study inclusion was that the patient is willing to try AM therapy. Possibly this willingness could in itself be associated with a more favourable prognosis, which might explain some of the observed improvement. Another consequence of the prerequisite of willingness is that study results apply only to patients who are willing to try AM therapies.

Adjunctive treatment with antidepressants or psychotherapy cannot explain the outcomes of our study, because symptoms improved similarly in patients not using such therapies. Other possible confounders are observation bias and psychological factors. Since, however, all AM therapies (including physician- and therapist-patient interactions) were evaluated as a therapy package, the question of specific therapy effects vs. non-specific effects (placebo effects, context effects, patient expectations etc.) was not an issue of the present analysis.

Since patients were treated by AM physicians who could possibly have an interest in AM therapies having favourable outcomes, the study data were largely collected by patients and not physicians. Any bias affecting physician's documentation would not affect CES-D (primary outcome), Symptom Score, or SF-36, since these clinical outcomes were documented by the patients.

Included in this study were outpatients aged 17–70 years with moderate to severe depressive symptoms. The patients were recruited by physicians offering routine care, and structured psychiatric interviews to assess all DSM-IV or ICD-10 depressive disorder criteria were not feasible, which limits diagnostic comparability with other studies. However, all patients fulfilled the DSM-IV core symptom criteria for dysthymic disorder and 82% of patients fulfilled the additional criterion of at least two years symptom duration.

Since AM was to be evaluated under routine conditions, therapy was not administered according to a standardised protocol but at the discretion of the physicians and therapists. Moreover, any of four AM therapies (AAT, EYT, RMT and MED) was permitted and the main analysis comprised all AM therapies. Subgroup analysis showed similar improvement of patients receiving EYT and AAT and in the AAT subgroup using painting/drawing/clay, but the sample size did not allow for analysis of RMT and MED groups nor of the other AAT technique subgroups.

### Study implications

This study provides the first data on AM therapy for depression in primary care. Notably, the female-to-male ratio was much higher in our study (5.5/1.0) than in other German primary care depression cohorts (1.3–2.6/1.0) [46-50]. A high proportion of women and of patients with higher educational levels, as observed here, has been observed in other studies of AM users [11,51,52]. Baseline depression severity in our study (CES-D average 35 points) was between the severity in untreated patients with Dysthymic Disorder (34 points) and Major Depression (39 points) [35]. Baseline SF-36 Mental Component Summary Measure (mean 26.2 ± 8.0) was slightly lower (median difference 0.34 Standard Deviations, range 0.30–0.72), i.e. worse, than in other primary care depression cohorts [53-56]. Altogether, our results suggest that primary care patients receiving AM therapy for depression are more frequently women, but resemble other depressed primary care patients regarding symptom severity and functional impairment. The higher proportion of women in this sample might possibly reflect that women are more likely than men to engage in artistic therapies like EYT and AAT.

In the first six months after enrolment, 55% of study patients had no standard therapy (psychotherapy, antidepressants) for depression. Some patients will not profit from standard therapies; other patients discontinue standard therapies due to adverse reactions or reject them because they are passive (antidepressants) or can be felt as intrusive or too verbal (psychotherapy). In this context, the AM non-verbal (AAT, EYT, RMT) and artistic exercising therapies (AAT, EYT) offer a different approach or even a bridge to opening up communication on a verbal level [11].

About one-third of enrolled patients improved by at least 50% of baseline CES-D scores and remained improved during the four-year follow-up; this rate is in the same order of magnitude as in long-term studies of psychotherapy for depression [7].

The results of this first study of AM therapy for depression in outpatients are encouraging and warrant further investigations.

## Conclusion

Among outpatients starting AM therapies for chronic depression, a large proportion continued in treatment and an encouraging proportion showed clinically relevant improvement. Although the pre-post design of the present study does not allow for conclusions about comparative effectiveness, study findings suggest that AM therapies can be useful for patients motivated for such therapies.

## Abbreviations

AM: anthroposophic medicine, AMOS: Anthroposophic Medicine Outcomes Study, AAT: AM art therapy, CES-D: Center for Epidemiological Studies Depression Scale, EYT: eurythmy therapy, IQR: interquartile range, MED: AM therapy (counselling, AM medication) provided by study physician, RMT: rhythmical massage therapy

## Competing interests

The author(s) declare that they have no competing interests.

## Authors' contributions

HJH, CMW, SNW, and HK contributed to study design. HJH, AG, and HK contributed to data collection. HJH, RZ, and HK wrote the analysis plan, HJH and AG analysed data. HJH was principal author of the paper, had full access to all data, and is guarantor. All authors contributed to manuscript drafting and revision and approved the final manuscript.

## Pre-publication history

The pre-publication history for this paper can be accessed here:


